# Investing in Food Safety for Developing Countries: Opportunities and Challenges in Applying Whole-Genome Sequencing for Food Safety Management

**DOI:** 10.1089/fpd.2018.2599

**Published:** 2019-07-09

**Authors:** Isabella Apruzzese, Eunyeong Song, Ernest Bonah, Vernadette S. Sanidad, Pimlapas Leekitcharoenphon, Julius John Medardus, Nagmeldin Abdalla, Hedayat Hosseini, Masami Takeuchi

**Affiliations:** ^1^ Franco Prattico Masters' Course in Science Communication, Trieste, Italy.; ^2^ Department of Chemistry, College of Chemistry and Chemical Engineering, Xiamen University, Fujian, China.; ^3^ Food and Drugs Authority, Northern Regional Office, Accra, Ghana.; ^4^ National Meat Inspection Service, Manila, Philippines.; ^5^ National Food Institute, Technical University of Denmark, Copenhagen, Denmark.; ^6^ Department of Veterinary Anatomy and Pathology, College of Veterinary Medicine and Biomedical Sciences, Sokoine University of Agriculture, Morogoro, Tanzania.; ^7^ Sudanese Standard and Metrology Organization, Khartoum, Sudan.; ^8^ National Nutrition & Food Technology Research Institute, Shahid Beheshti University of Medical Sciences, Teheran, Iran.; ^9^ Food and Agriculture Organization of the United Nations, Rome, Italy.

**Keywords:** whole-genome sequencing, next-generation sequencing, foodborne disease surveillance, food safety, developing countries, Food and Agriculture Organization of the United Nations

## Abstract

Whole-genome sequencing (WGS) has become a significant tool in investigating foodborne disease outbreaks and some countries have incorporated WGS into national food control systems. However, WGS poses technical challenges that deter developing countries from incorporating it into their food safety management system. A rapid scoping review was conducted, followed by a focus group session, to understand the current situation regarding the use of WGS for foodborne disease surveillance and food monitoring at the global level and identify key limiting factors for developing countries in adopting WGS for their food control systems. The results showed that some developed nations routinely use WGS in their food surveillance systems resulting in more precise understanding of the causes of outbreaks. In developing nations, knowledge of WGS exists in the academic/research sectors; however, there is limited understanding at the government level regarding the usefulness of WGS for food safety regulatory activities. Thus, incorporation of WGS is extremely limited in most developing nations. While some countries lack the capacity to collect and analyze the data generated from WGS, the most significant technical gap in most developing countries is in data interpretation using bioinformatics. The gaps in knowledge and capacities between developed and developing nations regarding use of WGS likely introduce an inequality in international food trade, and thus, relevant international organizations, as well as the countries that are already proficient in the use of WGS, have significant roles in assisting developing nations to be able to fully benefit from the technology and its applications in food safety management.

## Introduction

New foodborne pathogen analytical technologies often hold the promise of improving food safety, and whole-genome sequencing (WGS) is one of them (Deng *et al.*, 2016). This technology positively contributes to epidemiological investigations of foodborne outbreaks, to the identification of emerging health threats, to genome characterization of bacterial isolates, and to identify virulence, antimicrobial resistance (AMR), and other relevant genes in complex samples (Nadon *et al.*, 2017; Taboada *et al.*, 2017). As WGS evolves from a research tool to a practical food safety management instrument, a number of bioinformatic software and tools have been developed to enable seamless analyses of the sequenced foodborne pathogen data (Langmead and Nellore, 2018). WGS is already a routine tool to identify and characterize pathogens in developed countries (Jackson *et al.*, 2016), and significant amounts of relevant data have been stored systematically and the results of use of such data can be found on various websites, such as GenomeTrakr (US FDA, 2019).

New technologies are often perceived as too advanced by developing countries; some have stated that WGS is too sophisticated and data generated from the technology can be overwhelming as capacity development activities and trainings on rather basic microbiological analyses are still their fundamental needs (FAO, 2016). However, many researchers state that WGS itself is straightforward to perform, and this knowledge needs to be properly communicated (Allard *et al.*, 2016).

With the world population reaching a number of 9.1 billion in 2050 (UN/DESA, 2017), agricultural trade is expected to continue expanding (FAO, 2015). The global food supply chain has become extremely complex and it is not uncommon that one food item's ingredients come from various suppliers in multiple countries (McCullough *et al.*, 2008). This means even a single local contamination could affect a large portion of the food supply chain, thus affecting a large number of people on a global scale (Gharehgozli *et al.*, 2017).

In addition to the already problematic informal transportation of food items through smuggling and alike, the ever-increasing online food trade and direct e-food purchasing may create a sideline traffic of food items, which do not necessarily go through established border control systems (McCullough *et al.*, 2008). Consequently, it is increasingly necessary to strengthen food safety collaborations among countries regardless of their geographical location (Fukuda, 2015) and sharing data obtained through WGS has a potential in creating a functional global environment to enable such collaboration for effective foodborne disease outbreak management (Sasaki and Burr, 2000).

Incorporation of WGS into regulatory frameworks in developed countries has demonstrated its effectiveness in food safety management (Edmond-Rheault *et al.*, 2017), but whether WGS would be feasible and effective in developing countries needs to be assessed. A rapid scoping review, a keyword-based research synthesis that outlines the areas of research on a particular topic, was conducted followed by a focus group session to understand how realistically WGS can bring benefits to developing countries; how this technology can help improve the current situation; whether developing nations can meet essential requirements in terms of knowledge and infrastructures; and how introduction of WGS could affect international trade.

## Materials and Methods

A rapid scoping review was conducted using a standard framework (Arksey and O'Malley, 2005). A set of hypotheses was developed with primary, secondary, and tertiary keywords (Table 1). A series of searches were conducted using the various combinations of keywords in two bibliographic databases: ScienceDirect^*^ and PubMed.^†^ An additional search was conducted online for general publications and information. All searches were limited to publications/documents written in English and published up to July 2018. Keyword combinations were expressed using Boolean positional operators to refine the results (Spink *et al.*, 2001). To maintain the focus on WGS applications regarding microbiological hazards, the results on other types of contamination such as chemical and physical hazards were not considered.

**Table 1.** Scoping Review Keywords and Numbers of Relevant Hits

*No.*	*Primary keyword*	*Secondary keyword*	*Tertiary keyword*	*ScienceDirect*^a^	*PubMed*^b^
1	WGS	Outbreak		671	292
			Investigation	546	105
			Identification	486	59
			Detection	468	59
		Incident		678	5
			Investigation	544	
			Identification	272	2
			Detection	287	
		Case		11,530	202
			Investigation	8673	38
			Identification	3639	34
			Detection	3275	29
	NGS	Outbreak		833	89
			Investigation	633	19
			Identification	567	26
			Detection	614	25
		Incident		830	7
			Investigation	590	1
			Identification	357	
			Detection	406	1
		Case		17,554	815
			Investigation	11,478	37
			Identification	7455	145
			Detection	7777	146
2	Developing countries^c^	Microbial safety		21,019	97
			NGS	225	
			WGS	174	
		Outbreak		60,840	2627
			NGS	331	1
			WGS	375	1
		Food safety		99,188	1040
			NGS	433	1
			WGS	342	1
3	WGS	Data share		2990	10
			Trace	741	
			Routine surveillance	250	
			Transmission	761	1
	NGS	Data share		5608	27
			Trace	1080	
			Routine surveillance	95	
			Transmission	1194	3
	Genotyping	Data share		72,162	132
			Trace	10,255	1
			Routine surveillance	3670	1
			Transmission	19,277	19
4	Developing country	WGS		2337	8
			Source attribution	89	
			Interpretation	850	1
		NGS		2582	25
			Source attribution	66	
			Interpretation	917	3
5	Trade	WGS		1356	4
			Trace	350	
			Reference	962	1
			Frontline tool	4	0
			Phylogenetic	88	0
		NGS		1277	27
			Trace	289	
			Reference	888	1
			Frontline tool	12	0
			Phylogenetic	184	1
6	WGS	Prediction		3215	79
			Management	1128	6
			Risk assessment	621	1
		Forecast		609	1
			Management	366	0
			Risk assessment	197	0
7	WGS	Developing country		2337	8
			Retrospective investigation	170	0
			Microbiology investigation	305	0
			Epidemiology investigation	287	0

A rapid scoping review was conducted to outline the key thematic areas on the topic of WGS applications in food safety management. As the relevance was verified in the process, the higher number of hits indicates that the topic has been widely discussed in published literature and the lower number of hits indicates that the supporting evidence to the topic is not sufficiently available in published literature.

^a^ ScienceDirect. Available at: https://www.sciencedirect.com

^b^ PubMed—NCBI. Available at: https://www.ncbi.nlm.nih.gov/pubmed

^c^ Based on the UN country classification and terminology. Available at: https://www.un.org/development/desa/dpad/wp-content/uploads/sites/45/publication/WESP2018_Full_Web-1.pdf

NGS, next-generation sequencing; WGS, whole-genome sequencing.

Publications and information made available by key international organizations, including the United Nations (UN) specialized agencies, Organization for Economic Co-operation and Development and World Organization for Animal Health, were reviewed. Further review was conducted on the websites of some food safety competent authorities, including the Canadian Food Inspection Agency; European Centre for Disease Prevention and Control; European Food Safety Authority; United States Centers for Disease Control and Prevention; United States Food and Drug Administration; Food Safety Authority of Ireland; Food Safety Commission of Japan; United Kingdom Food Standards Agency; Food Standards Australia New Zealand; and Ministry for Food and Drug Safety in the Republic of Korea.

In order for the study to highlight the opportunities and drawbacks in incorporating WGS in countries with challenges in terms of both financial resources and technical capacities, the UN country classifications (UN/DESA, 2018) were used and the terminologies of “developed countries” and “developing countries” were applied.

Accordingly, a separate set of searches was conducted to find information on situations in developing countries and the sources included the following: National Service of Agri-Food Health and Quality in Argentina; the Ministry of Health and the Ministry of Agriculture, Livestock and Food Supply in Brazil; Agency for Quality and Food Safety in Chile; China Food and Drug Administration; National Food Safety Coordination Committee in Kenya; Ministry of Agriculture and Ministry of Health in Malaysia; Food and Drug Administration in Myanmar; Department of Agriculture in South Africa; and Department of Health and Department of Trade and Industry in South Africa.

To cover the possibility that the scoping review had an information gap regarding developing countries, a focus group session was conducted. Specific objectives of the focus group session were as follows: to confirm one of the key results of the scoping review that developing countries have not generally used WGS for food safety management; to assess the availability and accessibility of global WGS data; to collect feedback on specific challenges for developing countries in generating, storing, and sharing such data; to assess the capacity level for prerequisite activities necessary for use of WGS, such as isolating pathogens; and to identify the capacity needs in terms of infrastructure, knowledge, and technical capacities such as bioinformatics. Due to the geographical separation of participants, the focus group was conducted online.

Experts from Ghana, Iran, the Philippines, Sudan, Tanzania, and Thailand (*n* = 6) participated in the focus group session. All are members of the FAO informal network of developing countries to share information, knowledge, and experience in WGS (FAO, 2018). All confirmed their agreement to be recorded during the focus group session. The focus group questions were developed following a standardized pattern (Krueger, 2002). The recording was transcribed and analyzed with five stages: familiarization with the data, identification of thematic ideas, indexing of data, charting of quotes, and mapping and interpretation of data (Krueger and Casey, 2000).

## Results

The rapid scoping review successfully outlined key thematic areas of the topic, and as anticipated, the relatively low number of hits was compensated with the search strategy of repeating the combinations of synonyms of each keyword (Table 1). The scoping review identified that, compared with the previously confirmed number of countries (*n* = 4) that used WGS for food safety management in 2016 (FAO, 2016), more countries (*n* > 10) are using WGS for regulatory purposes. However, the review also confirmed that all those additional countries are developed nations. There were no data that any developing country had initiated using WGS in the government system and this was further confirmed by the focus group session with experts from developing countries.

The search combining keywords with the technology, “case” and “investigation” resulted in a high number of hits, indicating that many articles report the positive impacts that implementation of WGS has had in surveillance systems. Most of these reports describe how introduction of WGS has increased the number of detected outbreaks and surveillance (den Bakker *et al.*, 2014; Dallman *et al.*, 2015; Kwong *et al.*, 2015; FAO, 2016; Jackson *et al.*, 2016). Nonexperts may wrongly perceive that introduction of WGS is creating more food safety problems rather than solving them, but experts state that the technology brings major public health benefits by minimizing the scale of the outbreaks and preventing recurrence of the problem from the same source (Kwong *et al.*, 2015).

In general, the scoping review generated a very low number of hits for any searches with the keyword “developing countries,” indicating that this type of information may not be usually reported in peer-reviewed journals. There is some evidence that WGS is being introduced at the research level in various developing countries and many scientists have good understanding of its potential (Healy *et al.*, 2016). Some research institutes, such as Institut Pasteur, have conducted WGS-related studies in developing countries to promote the technology (Weill *et al.*, 2019).

During the focus group session, the most consistent findings were that scientific knowledge, understanding of potential applications and benefits, and strong motivation to use WGS exist, but they expressed an immediate need to increase the level of understanding by high-level government officials. Therefore, it can be concluded that a systematic incorporation of WGS into the government systems in developing nations has not yet occurred, and thus, the technology still is not used as an official food safety management tool (Pekdemir, 2018). Understanding both the reality and the perceptions from experts in such developing countries could significantly contribute to the formulation of a global strategy on WGS and food safety (Pekdemir, 2018). Table 2 presents the summary of the focus group results.

**Table 2.** Summary of the Focus Group Results

*No.*	*Topic*	*Responses*
1	Use of WGS for official food safety management activities	• WGS is not being used for food safety management in any countries that participants are from.^a^ • Priority conflict exists as many countries focus on food security. • Awareness of the potential benefit of WGS in the area of food safety is low.@ • No budget to consider the new technology to be employed in the area of food safety.
2	WGS data collection and accessibility to the global database	• No national database of WGS for food safety exists in any countries participants are from. • All were aware of the global databases that they can access, but the access from the government sector is extremely limited. • Two countries have genomic center and relevant research institute that can host WGS data for food safety, but currently no accessible data are available.
3	WGS data generation	• Three participants are involved in WGS data generation and the data are shared on some global databases. • There is a recognition that WGS data generation from countries such as theirs would be useful in strengthening their surveillance systems. • Participants stated that currently there are no awareness and/or interest in the government sector to generate WGS data for food safety management.
4	Food safety data sharing	• Some countries share food safety-related data for research purposes. • AMR data are proactively shared on global databases. • Participants are aware of some data-sharing platform for food safety, such as the INFOSAN, but active data sharing from their countries with international community is rare.
5	Basic laboratory capacity	• Participants confirmed that their countries have reference laboratories that can isolate pathogens, thus basic microbiological capacity exists. • The work is not currently aimed at application of WGS.
6	Food and environmental monitoring for foodborne pathogens	• All participants confirmed that clinical samples are often checked on a regular basis, but food and environmental monitoring for foodborne pathogens is usually done on an *ad hoc* basis, when some incidents are suspected. • Budgetary constraints exist to regularly conduct such activities.
7	Technical challenges to improve food monitoring systems	• As food safety is a cross-cutting issue, communication and collaboration with different sectors (agriculture, health, trade and commerce) are a challenge. • General awareness on the importance of microbiological food safety issues is low. • Financial and human resources are limited.
8	Bioinformatic capacity	• Capacities exist at the research level, but all participants confirmed that the capacity is not sufficient at the government level. • Some countries confirmed that bioinformatics is not in the curricula of major universities, and thus, the number of bioinformaticians in their countries is very low.
9	Needs in improving WGS knowledge and capacity	• There is a strong need in raising the awareness of the potential of WGS for food safety in developing countries. • The focus for knowledge and capacity improvement should be in the government and regulatory sectors. • A long-term vision is necessary to initiate the awareness campaign to follow the technology development and trend in food safety analysis.
10	Potential of WGS in developing countries	• Participants strongly agreed that WGS would increase the number of detected food safety incidences and outbreaks. • If the number of outbreaks is properly reported, food safety will become a priority for their countries, and thus, the technology would contribute to protecting public health.

A focus group session was conducted with technical experts (*n* = 6) from developing countries to assess the current situation regarding WGS applications in food safety management for developing countries. Based on a standard pattern of analyzing a focus group study, the items in the left column represent thematic ideas and the items in the right column represent the indexed and mapped interpretations of quotes.

^a^ Ghana, Iran, the Philippines, Sudan, Tanzania, and Thailand.

AMR, antimicrobial resistance; INFOSAN, International Food Safety Authorities Network; WGS, whole-genome sequencing.

The scoping review also had a relatively low number of hits when searching about the food safety situation in developing countries, which implies that developing countries are currently facing significant challenges in detecting foodborne outbreaks and/or in reporting outbreaks in publishable articles (Ahmed *et al.*, 2015; Odeyemi, 2016). Assuming that it is already costly for developing countries to fully implement traditional methodologies to detect foodborne outbreaks, it is necessary to question whether incorporating WGS would be cost-effective in improving outbreak detection/reporting situations.

Conventional methodologies to detect foodborne pathogens often require laboratories to obtain an official accreditation for species-specific identification and typing protocols. The universality of WGS has a benefit in efficiency and the cost-per-sample has been significantly decreasing. Although WGS could contribute to cost savings for identification of foodborne pathogens, the overall cost may still be high as WGS requires relevant infrastructures and functioning equipment/personnel. In addition, the cost-per-run of sequencing is still perceived as costly in many countries (FAO, 2016). The issue of WGS cost being both a benefit and a drawback for developing countries has been also documented in detail in a previous study (FAO, 2016).

Another challenge that developing countries face when considering use of WGS for foodborne disease surveillance is regarding data handling and sharing. WGS generates a large amount of data, characterized by a high volume of information, high-speed production of data, and a wide variety of extremely precise information—all of which are useful for risk managers to strengthen their understanding of the complexity and diversity of a food safety problem (Bergholz *et al.*, 2014). However, significant data mining efforts are necessary to attribute the source of transmission of a foodborne pathogen and provide insights on control strategies (Langmead and Nellore, 2018), and such expertise in bioinformatics is not readily available in developing nations (Pongor and Landsman, 1999).

A number of user-friendly cloud-based tools are now available that can be used by people without formal bioinformatic education. Use of these tools may alleviate availability of bioinformaticians as a critical need for developing countries (NCBI, 2018) and (Fierro *et al.*, 2018).

WGS-generated data can be especially important for international food trade (Zankari *et al.*, 2013). While only a limited amount of information on the status of WGS incorporation in developing countries was available in literature, many industrialized nations have already incorporated this technology in their food safety regulatory framework. The imbalance in use of WGS may pose compliance difficulties to the developing countries who often export food products to developed countries (Franz *et al.*, 2014).

Application of WGS would reduce the need of multiple tests at the borders (Yoshida, 2014) and its accuracy, together with its timely results, is likely to make a positive impact on international food trade in the future (Zankari *et al.*, 2013). However, if this technological advancement is only happening in developed countries, it may create inequality in trade. Many developed countries have been incorporating new approaches and new technologies to upgrade their national food control systems^‡^ in the last decade (Gilchrist *et al.*, 2015), and as a consequence, a possibly large gap between developed and developing nations might have been induced. Such a gap indirectly imposes the pressure to some exporting developing countries in needing to increase their technical capacities to the ones of the importing developed countries (Veggeland and Borgen, 2005).

A number of articles explore the predictive power of WGS data (Gordon *et al.*, 2014), demonstrating that its use in outbreak investigations can support decision makers and therefore can reduce the misidentification of food sources (Parkhill *et al.*, 2000). However, the scoping review revealed that this advantage has not been discussed much in developing nations. Also, since there were almost no bioinformatic-related studies found in the scoping review on WGS and food safety in developing countries, it can be concluded that there may not be a sufficient number of bioinformaticians available in developing countries. To sustain effective national food control systems where WGS data are regularly used, many bioinformatic-related challenges must be addressed in developing countries (Fricke and Rasko, 2014).

Results of the focus group session provided remarkable information that these experts from developing countries are confident about WGS and that scientific capacity to develop the technology exists. All participants demonstrated awareness about the potential of the technology, some of them have had extensive experience with it, and two of the countries represented by those interviewed currently operate genomic research centers.

The main issue they are facing is connected to priority conflicts that result in national governments not dedicating financial/human resources for the introduction of WGS. All focus group participants stated that they are more than ready to use the technology to contribute to their national food control systems, but lack of commitment at higher levels of government has been a barrier to their use of WGS. To fill the gap, knowledge, and capacity, collaboration between developed and developing countries and technical assistance from international organizations are needed. However, factors such as the current movement toward proactive global sharing of AMR data, awareness of experts about WGS, and existence of university degree programs in bioinformatics in some developing countries, together with the high level of interest and strong motivation expressed by focus group participants, suggest developing countries are getting ready to introduce WGS.

## Discussion

WGS is a powerful tool to identify and characterize foodborne pathogens, and if routinely used, it can prevent and control outbreaks (Joensen *et al.*, 2014). Currently more than 10 countries use the technology for food safety management, making WGS an essential tool to more completely understand food microbiology (Allard *et al.*, 2018). As of July 2018, between 11,000 and 18,000 scientific articles discuss the use of genomic technologies for identification, investigation, and/or prevention of foodborne disease outbreaks. In fact, one of the major advantages of WGS is the speedy subtyping of foodborne bacterial pathogens worldwide (Nadon *et al.*, 2017).

A few case studies from the United States, Denmark, and England have shown how WGS can be incorporated into the food safety regulatory system for outbreak investigations with benefits, such as specificity, allowing improved case definition to enhance the outbreak management; sensitivity, enabling linkage of apparently sporadic diseases occurring under the outbreak surveillance radar; and precision, determining the root cause of complex outbreaks (FAO, 2016).

The major success of WGS is that it enables sharing of high-resolution data of the genomes, giving the possibility to identify genetic variants, and to explore the effects of gene expression and regulation (Gilad *et al.*, 2009). In addition, the large quantity of high-quality data produced in a short amount of time makes this technique efficient as well as accurate (Daetwyler *et al.*, 2014). Latest sequencers are equipped with a function to combine many steps, including template preparation, sequencing, imaging, genome alignment, and assembling the sequenced data, making the laboratory procedures drastically simple and fast (Metzker, 2009).

Hence, WGS has positively contributed to timely and effective foodborne outbreak investigations in those countries that utilize the technology, confirming that it will be an excellent “next-generation” tool that will play a significant role in the area of food safety (Wang *et al.*, 2016). However, many developing countries may not agree that it is efficient and inexpensive in their circumstance. Countries without established surveillance systems may not see the cost/benefit of adding WGS capability and implementation of WGS may divert essential resources from more pressing priorities (FAO, 2016).

Underreporting of foodborne diseases and microbial contamination cases is common in both developed and developing countries; however, the extremely low number of outbreaks reported by developing nations likely does not reflect the actual situation in these countries, especially if compared with the large numbers of outbreaks reported on a regular basis by many developed countries (Fierro *et al.*, 2018) as well as with the estimated global burden of foodborne diseases (WHO, 2015). A major reason for this extreme underreporting in developing countries is often linked to limited capacities in their national food control systems with insufficient resources and infrastructures (Grace, 2015).

The availability of diagnostic services to report infections is also key, because without it countries have to rely only on syndromic surveillance, without sufficient sensitivity and specificity. Proper implementation of internationally harmonized measures and regulations (WHO, 2005) might help developing countries address this problem. Before the introduction of WGS, it is essential for countries to have a systematic mechanism to collect isolates and their metadata from both clinical samples and food/environment samples (EFSA, 2008; FAO, 2016). Unless these matters are addressed, introduction of WGS will remain a tool used in research projects only.

While the focus group participants declared that well-equipped in-country laboratories are available for analysis of isolates, WGS analysis needs to be conducted routinely if it is used as the basis for food surveillance systems. Therefore, assessing the capacity in developing countries to confirm the feasibility in establishing WGS-based surveillance systems for both food supply and clinical infections is important, taking into consideration the current isolate and data collection mechanisms (FAO, 2016). However, a question remains whether the unavailability of a complete surveillance system should stop integration of WGS in developing countries—and the focus group participants suggested that the opportunity is to be presented equally to anyone to advance knowledge. Also, Helmy *et al.* (2016) discussed that, in their study in developing countries, health risk identification, diagnoses, treatment, and prevention would likely be improved with new tools and technologies.

Improvement of pathogen detection capacity could lead countries to have a food safety monitoring situation that more accurately reflects the reality of their situation to undertake food safety surveillance activities. For example, the introduction of WGS in Kenya drew attention of decision makers to the overall importance of food safety and thus provided the basis for the development of a national food control system (FAO, 2016). The Kenya Medical Research Institute introduced the use of WGS to sequence strains from selected pathogens from clinical samples. With the detailed data obtained through WGS, the Kenyan Government was able to map disease hotspots to revise existing treating regimens and identify high-risk foods. As a result, interest in investing more on food safety was increased and the usefulness of WGS was recognized for analysis of microbial food contamination for regulatory interventions (FAO, 2016).

WGS is innovative because it does not require targeting primers (Burall *et al.*, 2017), but it allows molecular subtyping (Bal *et al.*, 2016) and further state-of-the-art analyses using sophisticated approaches (Kase *et al.*, 2017). Moreover, WGS has shown to provide advantages, compared with traditional methods, because of its suitability to identify AMR and to detect the emergence of new foodborne pathogen strains (Baker *et al.*, 2017). AMR is already a global concern (Paterson, 2006) and WGS can accurately identify resistant genotypes and predict resistant phenotypes (Tyson *et al.*, 2015). Resistance levels have increased in pathogens that are more common in developing countries, and this trend is increasing (Okeke *et al.*, 2005).

Many authorities in developing nations have shown interest in addressing AMR, because their countries show increasing numbers of cases of AMR-associated illnesses (WHO, 2001). When developing countries utilize WGS for microbiological risk assessment related to AMR, it can also benefit food safety management as food is one of the major vehicles of AMR (FAO/WHO, 2018). The topic of AMR was spontaneously raised by the focus group participants who agreed that the accuracy of WGS, in particular, the application of single-nucleotide polymorphism-based AMR identification, could play a key role in addressing the problem.

While WGS holds technological advantages, the data have to be interpreted, stored, and shared to make a comparison with data from other sources (Bergholz *et al.*, 2014). Results obtained from sequencing will only be useful for interpretation after they are put into bioinformatic databases (Franz *et al.*, 2014). Therefore, not having access to relevant comparable global data makes the outputs obtained through WGS almost useless. Such restriction is partially due to IT-related issues that involve high-capacity computers (Karunaratne *et al.*, 2018) or fast, reliable internet connections (Karunaratne *et al.*, 2018), which constitute a serious problem in developing countries and without which even easy-to-use, online bioinformatic tools (NCBI, 2018) cannot be used.

The availability of high-speed wireless internet connections such as 5G is expected to address this issue in most developing countries, but the full benefit of this may be realized several more years later. Moreover, lack of international standards regarding the quality of the sequenced data represents another important issue that needs to be solved. In addition, if there is no previously sequenced genome on which to perform the bioinformatic alignment, the identification process through WGS is neither simple nor fast (Yang *et al.*, 2017).

A number of countries have established WGS data-sharing mechanisms and there are some key databases and platforms developed for routine use. These include the US FDA GenomeTrakr (Allard *et al.*, 2016), which is a subset database of the Sequence Read Archive at the National Center for Biotechnology Information (NCBI), the European Nucleotide Archive, and the deoxyribonucleic acid (DNA) Data Bank of Japan (FAO, 2016). Although accessibility to the real-time global databases is highly desirable, microbial evolution makes it hard to obtain up-to-date, complete information, since only a small fraction of microbes will ever be cultured and sequenced (Selifonova *et al.*, 2001). Even more challenging is the reluctance of the countries to share their data in real time.

Some initiatives seek to facilitate global data sharing: a scientific consortium, the global microbial identifier (GMI) envisions a global system of DNA genome databases for microbial and infectious disease identification and diagnostics (GMI, 2018); an international laboratory network, the PulseNet International works toward the standardized use of WGS methods and data to identify and subtype foodborne bacterial pathogens worldwide (Nadon *et al.*, 2017); and GenomeTrakr provides an open source WGS database for microbial pathogens collected and publically shared by agencies in real time (US FDA, 2018). These initiatives are supported by technical experts from many countries and Taboada *et al.* (2017) stated that more effort needs to be made to develop or unify a global tool to make relevant WGS data as the one-of-a-kind interoperable resource.

None of the countries involved in the focus group session has its own national WGS database. Some foodborne disease-related data exist, but the data are currently being obtained through traditional techniques; they are often not systematically collected and are only occasionally published in scientific journals. Scientists in developing countries use the abovementioned global databases to publish their data, but the reports do not constitute a national data set, because there is no system to consolidate them. Further work needs to be done in order for such countries to systematically use WGS as an integrated part of their national food control systems.

International market trends demonstrate that there are many opportunities for developing countries in export of food products (Henson and Jaffee, 2006). These markets are driven by competition based on safety and quality of products, and therefore, the associated characteristics are codified in standards (Ponte and Gibbon, 2005). Such standards can act both as a barrier and a catalyst to upgrade food management capacities, because they can facilitate or constrain the access of developing nations to high-value markets for agricultural and food products (Henson and Jaffee, 2006). Use of WGS data to assay for foodborne pathogens creates trade opportunities for developing countries and can be advantageous for them to meet the quality assurance criteria requested by industrialized countries (Henson and Jaffee, 2006). Moreover, WGS data could be used as a supportive evidence for decision makers in case of food trade disputes.

To mitigate the food safety challenges associated with food trade, a global collaborative effort is required (Buzby, 2003). For example, an economical estimation study on possible harmonization for a standard on aflatoxin B1 in nuts and cereals showed a potential increase of world exports by USD 38.8 billion for nuts and USD 6.1 billion for cereals compared with total exports when using divergent national standards (Wilson and Otsuki, 2001). This study further revealed that in addition to the gain in safety of the products, a healthy global competition would likely promote fair practices in global food trade. Technical assistance to developing countries provided by neutral international organizations would help them effectively access international markets in a proactive way (Henson and Jaffee, 2006).

The potential economic benefit of using WGS for detection of contaminated sources is worthwhile to discuss further with developing countries. Food safety problems often result in large-scale financial losses due to product recalls, disposals, and penalties, along with potential damage to the overall reputation of food producers, companies, and countries, with the resulting decrease in consumers' confidence (Hussain and Dawson, 2013). In theory, use of WGS with its precision could contribute in avoiding unnecessary food recalls due to possible source-attribution mistakes and even eventually contribute in reducing food waste. The specificity and sensitivity of WGS could provide more targeted approaches to regulatory authorities (FAO, 2016), and thus, WGS can become a good tool to reduce the economic burden for developing countries.

Developing countries may need feasibility studies to assess their priorities, their requirements, and their readiness to incorporate WGS in food safety management (FAO, 2016). In particular, WGS may not be the most effective tool in the situation where food safety policies or national food control systems do not function well. Technical capacities to manage the technology and relevant tools of WGS will eventually be needed, but to assure implementation of WGS, government officials who have an understanding of how WGS works are a key factor (Healy *et al.*, 2016). Developed nations need to pay attention to the emerging needs of developing countries because the challenges the latter has to face may have been imposed by the richer nations (Henson and Jaffee, 2006). Major benefits for all could arise from assuring equal opportunities and having globally comparable WGS systems (Sansone *et al.*, 2012).

## Conclusion

Results from the scoping review and the focus group session indicated that in developing countries, WGS is rarely used in nonresearch settings. Governmental authorities do not always see food safety as a priority, and thus, they do not invest in tools such as WGS to test foods for contamination or to investigate foodborne diseases.

The gap in capacity between developing and developed countries when applying WGS may create inequalities in ensuring fair food trade. In developing nations, infrastructure and technical capacities, including laboratory conditions and bioinformatic capacities, still constitute a challenge to realistically apply WGS (Ahmed *et al.*, 2015). This can be of a global concern because the core nature of WGS largely lies in data sharing to have a real-time access to global data to match with the relevant local data sets (Kaye *et al.*, 2009) and partial collection of data from limited geographical areas will not reach the maximum results that WGS could provide.

Collecting and globally sharing such data in a comparable format benefit all to have a better food safety situation from the angles of both public health and global food trade (Pekdemir, 2018). In order for all countries to benefit from WGS, the work of various global initiatives aiming at building knowledge around WGS-related capacities, such as GMI, GenomeTrakr, and PulseNet, will be essential.

Timely scientific advice provided by international organizations to facilitate knowledge transfer and discussions on global data management is important to ensure the technology will benefit all. Technical support to developing nations is the immediate need to be provided by international organizations, experienced countries, and global technical alliances with capacity development activities focusing on practical aspects of WGS application in food safety management.

## Notes

^*^ http://www.sciencedirect.com

^†^ http://www.ncbi.nlm.nih.gov/pubmed

^‡^ Codex Alimentarius Commission provides principles and guidelines for national food control systems (CAC/GL 82-2013. Available at: http://www.fao.org/input/download/standards/13358/CXG_082e.pdf) and the terminology of “national food control systems” has been widely used among food safety competent authorities worldwide to indicate the overall food-related systems to protect the health of consumers and ensure fair practices in the food trade.
